# *SMPX* Deficiency Causes Stereocilia Degeneration and Progressive Hearing Loss in CBA/CaJ Mice

**DOI:** 10.3389/fcell.2021.750023

**Published:** 2021-10-14

**Authors:** Hailong Tu, Aizhen Zhang, Xiaolong Fu, Shiqi Xu, Xiaohui Bai, Haibo Wang, Jiangang Gao

**Affiliations:** ^1^School of Life Sciences, Shandong Provincial ENT Hospital, Shandong University, Jinan, China; ^2^University of Edinburgh Institute, Zhejiang University School of Medicine, Zhejiang University, Haining, China; ^3^Otolaryngology-Head and Neck Surgery, Shandong Provincial ENT Hospital, Jinan, China

**Keywords:** *Smpx*, hair cell, stereocilia degeneration, hearing loss, CRISPR-Cas9

## Abstract

The small muscle protein, x-linked (*SMPX*) encodes a small protein containing 88 amino acids. Malfunction of this protein can cause a sex-linked non-syndromic hearing loss, named X-linked deafness 4 (DFNX4). Herein, we reported a point mutation and a frameshift mutation in two Chinese families who developed gradual hearing loss with age. To explore the impaired sites in the hearing system and the mechanism of DFNX4, we established and validated an *Smpx* null mouse model using CRISPR-Cas9. By analyzing auditory brainstem response (ABR), male *Smpx* null mice showed a progressive hearing loss starting from high frequency at the 3rd month. Hearing loss in female mice was milder and occurred later compared to male mice, which was very similar to human beings. Through morphological analyses of mice cochleas, we found the hair cell bundles progressively degenerated from the shortest row. Cellular edema occurred at the end phase of stereocilia degeneration, followed by cell death. By transfecting exogenous fluorescent Smpx into living hair cells, Smpx was observed to be expressed in stereocilia. Through noise exposure, it was shown that Smpx might participate in maintaining hair cell bundles. This *Smpx* knock-out mouse might be used as a suitable model to explore the pathology of DFNX4.

## Introduction

X-linked hearing loss accounts for 1-2% of all non-syndromic hearing loss (NSHL) cases. Five genes have been associated with x-linked hearing loss, including *PRPS1* for DFNX1, *POU3F4* for DFNX2, *SMPX* for DFNX4, *AIFM1* for DFNX5, and *COL4A6* for DFNX6^1^.

Mechanosensory hair cells are primary cells that enable mammals to sense and distinguish sounds with different frequencies and intensities. These cells, located in the inner ear, can convert sound-induced vibrations into electrical signals. The inner ear can responds to sound-induced vibrations of less than a nanometer and amplify signals by more than 100-fold, and has a wide dynamic range of hearing enabling humans to perceive frequencies from 20 Hz to 20 kHz (Schwander et al., 2010).

Outer hair cell (OHC) bundles are composed of 3 height-ranked rows of stereocilia-modified microvilli packed with highly cross-linked actin filaments (Corvino et al., 2018; Qi et al., 2019, 2020; Liu Y. et al., 2019). Stereocilia, the mechanosensory actin protrusions on the roof of hair cells, play a key role in the transduction of vibration into electrical signals (Wang et al., 2017; Zhu et al., 2018; Cheng et al., 2021). The vertebrate auditory epithelium displays a gradient of stereocilia bundles, the lengths of which is inversely proportional to the frequency of the sound detected by cell tuning (Crawford and Fettiplace, 1985; Howard and Hudspeth, 1987; Ricci et al., 2000; Petit and Richardson, 2009). Although stereocilia are extremely sensitive to mechanical vibration, they are easily damaged by over-stimulation, such as sharp, high-decibel sound. Stereocilia share many construction principles with the formations of actin in microvilli and filopodia. Yet, the length of different stereocilia in a single cell can vary from 1 up to 120 μm and persist for a lifetime. The length of stereocilia may be influenced by actin treadmills, cross-links within the actin core, plasma membrane tension, interstereociliary links, and the overlying extracellular structures (Manor and Kachar, 2008). The turnover of actin filaments has long been considered to reflect the treadmilling behavior. However, recently studies have identified that filament barbed ends can be used for both elongation and disassembly (Romet-Lemonne and Jégou, 2021). In these aspects, actin binding proteins (ABP) are the main parts regulating the length of stereocilia.

The small muscle protein, x-linked (*SMPX*), originally identified and mapped in Patzak et al. (1999) is abundantly expressed in heart and skeletal muscles. In muscle cells (Kemp et al., 2001; Palmer et al., 2001) SMPX has an important function in protecting the sarcolemmal plasma membrane from mechanical stress. In Huebner et al. (2011); Schraders et al. (2011) proposed *SMPX* as the causative gene to DFNX4 in two Dutch families, one German family, and one Spanish family. Palmer et al. (2001) found that *Smpx*-null mice had no obvious developmental defects in the heart or skeletal muscle, which indicated functional redundancy. However, after the identification of hearing loss-associated gene *SMPX*, there are no detailed reports on how this gene causes hearing loss *in vivo*. In this study, we established a progressive hearing loss mouse model *via* CRISPR-Cas9 (Jinek et al., 2012; Cong et al., 2013), which is highly consistent with the DFNX4 displayed in humans.

## Materials and Methods

### Information Collection on Hearing Loss in Two Chinese Families

Data on individual patients were gathered and provided by the Institute of Otolaryngology, West Hospital of Shandong Provincial Hospital. NGS sequencing and data analyses identified three heterozygous mutations that were potentially pathogenic. After that, all exons and intron/exon boundaries containing or surrounding *SMPX* mutations were PCR-amplified using specific primers. The protocols for PCR reaction and amplification were described in a previous study (Zhang et al., 2016, 2018). Sanger sequencing of *SMPX* mutations was performed using the following primers: forward 5′-GCTTATGGCCCAAAG AGATC-3′ and reverse 5′-GGCCCAAAAACTTGGCTTAAC-3′. *SMPX* mRNA (RefSeq NM_014332) was used as a reference to align the sequences using Lasergene-SeqMan software.

Phylogenetic analysis of *SMPX* was performed with multiple sequence alignment using Clustal Omega software. The sequences included NP_055147.1 (Homo sapiens), XP_003317426.1 (Pan troglodytes), XP_001086790.2 (Macaca mulatta), NP_445847.1 (Rattus norvegicus), NP_001032715.1 (Bos taurus), XP_854567.1 (Canis lupus), and NP_001239520.1 (Mus musculus).

### Generation of *Smpx* Knockout Mice

*Smpx* knockout mice were generated in the animal facility of School of Life Sciences, Shandong University. Animal management was performed in strict accordance with the standards of the Animal Ethics Committee of Shandong University. All animals were housed in a quiet environment with a temperature of 22 ± 1°C, relative humidity of 50 ± 1%, and a light/dark cycle of 12/12 h.

Gene knockout mice were generated using the CRISPR-Cas9 gene-editing system. To avoid aging-related hearing loss resulting from Cdh23 single nucleotide substitutions carried by C57BL/6 mice, CBA/CaJ mice were used instead. CRISPR/Cas9 genome editing in mice was performed as previously described (Shen et al., 2013). PUC57-sgRNA expression vector containing single guide RNA scaffold (Addgene ID: #51132) and pST1374-NLS-flag-linker-Cas9 containing humanized Cas9 (hCas9) (Addgene ID: #44758) were obtained from Addgene. Two pairs of primers (Table 1) were used to amplify sgRNA transcription templates. Target sequence (20 bps) was directly cloned in the forward primer to avoid the enzyme digestion process. The vector of pST1374-NLS-flag-linker-Cas9 was linearized by SpaI. Cas9 mRNA and SgRNA were synthesized *in vitro* using AM1345 (Ambion) and AM1354 (Ambion).

**TABLE 1:** The primers used in KO mouse generation.

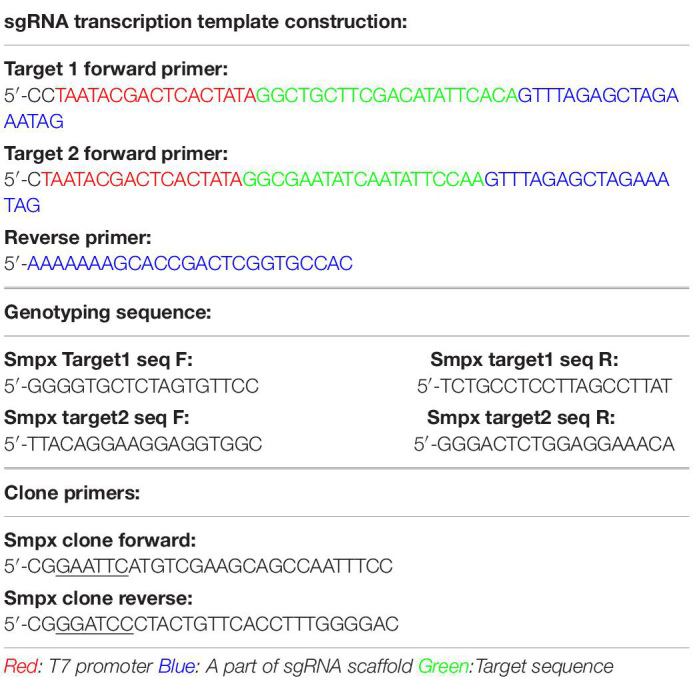

CBA/CaJ female mice were superovulated and mated with CBA/CaJ male mice. Fertilized eggs were isolated from the oviducts of the female mice. Mixed RNAs were then microinjected into the pronucleus and cytoplasm of the fertilized eggs under an inverted microscope. The injected fertilized eggs were incubated in KSOM-AA medium (Millipore) for ten minutes and then transferred into the oviducts of pseudopregnant CD1 female mice.

To identify the mutant mice, genomic DNA was extracted from the toes of pups, and a DNA fragment surrounding the target site was PCR amplified. PCR products were subjected to sequencing or cloned using the T/A cloning kit and then sequenced.

### Analysis of Off-Target Mutations

Predictable off-targeting sites were found by using Off-Spotter^2^. Five potential sequences and corresponding amplification primers were shown in Table 2. To reduce the impact of off-targeting, *Smpx* null mice were consecutively outcrossed with wild-type CBA/CaJ mice for five generations.

**TABLE 2:** The potential off-target site and their amplification primers.

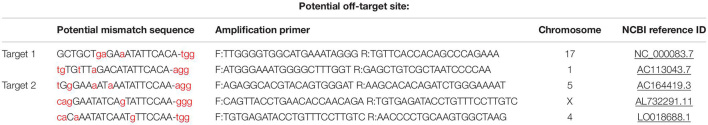

*Red color means the mismatched sites.*

### Auditory Brainstem Response and Distortion Product Otoacoustic Emission

Auditory brainstem responses (ABRs) of wild type, heterozygous, and homozygous -null mice were recorded from 1 to 5 months after birth. Mice were anesthetized with 0.007 g/ml sodium pentobarbital and placed in a soundproof box. All ABR recordings were captured by three subdermal needle electrodes. The positive electrode was inserted subcutaneously into the midline of vertex, the negative electrode was into the mastoid of the right ear and the ground electrode was into the rear region above the tail. ABR tone pips at frequencies of 4, 8, 16, and 32 kHz were generated using a Tucker Davis Technologies (TDT) workstation running the SigGen32 software and ABR data were recorded using the BioSig32 software. The stimuli were created and equalized with the audio editing software Audacity Portable. The ABR threshold was defined by a visual detection of the third peak in the waveform for click stimulus and at least one of the waves in response to the tone stimulus. The sound intensity was decreased from 90 to 20 dB until the lowest response waveform could not be identified.

Statistical analyses were performed using the GraphPad Prism 9 software. Different threshold groups collected in ABR were compared using the two-way ANOVA, followed by Fisher’s LSD test. A total of 90 mice were used in these experiments (*n* = 12 in 1 month WT and KO male mice groups; *n* = 11 in 3 months male WT and KO male mice groups; *n* = 8 in 5 months WT and KO male mice groups; *n* = 7 in all female mice groups). Mean values and standard deviation(SD) were used to describe the variability. A *p*-Value of <0.05 indicated statistical significance. Cohen’s d with Hedges’ g correction was used to calculate the effect sizes of ABRs, which reflected the influence of variables such as knock-out processing.

At 24 h following ABR recordings, DPOAEs at 2f1-f2 from mice that were anesthesized in the way above were obtained using the Real-time Signal Processing System III from Tucker-Davis Technologies. The primary tone produced by two separate speakers was placed as a combination microphone/speaker system in the animal’s sealed ear canal near the tympanic membrane. The DPOAE recordings were made with a low-noise microphone. All stimuli were digitally synthesized using TDT SigGen32 software. Primary tone frequencies (f1 and f2) differed by a factor of 1.25. The test frequencies were at 4, 8, 12, 16, 24, and 32 kHz and the levels were reduced in 10-dB steps from 80 to 20 dB. A fast Fourier Transform (FFT) was applied to obtain the magnitude of the 2f1-f2 distortion product. A peak at 2f1-f2 in the spectrum was accepted as DPOAE. Four WT mice and five KO mice of 3 months were used in this experiment.

### Immunostaining of Cochlea Basilar Membrane Stretching Preparation

Cochleae of wild-type and *Smpx* KO mice were isolated from mice aged 1, 3, and 5 months (3 mice each group). In each cochlea, a part of the cupula cochlea was removed. Paraformaldehyde (4% in PBS) was gently perfused into the cochlea through the opening. Then, the entire cochlea was immersed in a fixative solution for 2 hours at room temperature, followed by washing with 10 mM PBS. After that, the cochleae were decalcified in 10% EDTA overnight at 4°C. Softened cochleae were dissected into three or four parts of the basilar membrane, including the apical, middle, and basal turns. After 3 washes by PBS, samples were blocked with goat serum at 37°C for an hour and then stained with anti-myosin VIIa polyclonal antibody (rabbit, Proteus-Bioscience) overnight at 4°C. After that, the samples were incubated with secondary antibodies for 1 h at 37°C, followed by washing in PBS. Finally, samples were stained with rhodamine-phalloidin (Sigma) and DAPI (Sigma), followed by a final wash in PBS. Sections were visualized under an LSM 700 confocal microscope.

#### FM1-43 Uptake Assay

The electromechanical conversion function of OHCs mainly depend on MET Channel on OHC bundles. To examine if there were any changes of MET channel on degenerated OHC bundles, FM1-43 uptake was carried out. FM1-43, a fluorescent dye that permeated the transduction channels, was used as a preliminary exploration of MET channel. The FM1-43 uptake assay was performed as previously described (Lelli et al., 2009). Cochleae were dissected from wild-type and *Smpx*-null mice, and basilar membranes were acquired following the same protocol explained in the immunostaining assay. Samples were treated with 2 μmol FM1-43 dye (Invitrogen) in PBS for 20 s and then fixed with 4% PFA overnight at 4°C. After three times washing in 10 mM PBS, the sections were visualized under an LSM 700 confocal microscope. Seven middle turn areas were captured in each group. The fluorescence intensity was calculated by ImageJ software and the relative fluorescence units were used to represent the results.

### Scanning Electron Microscopy

Under sodium pentobarbital anesthesia, wild-type (5 male and 2 female) and *Smpx* KO mice (7 male and 4 female) were transcardially perfused with 4% PFA. The cochleae were isolated and immersed in 2.5% glutaraldehyde for 10 hours at 4°C. In order to soften the tissue, the cochleae were decalcified in 10% EDTA overnight at 4°C. The basilar membranes were dissected from softened cochleae and post-fixed in 1% osmium tetroxide (diluted by ddH_2_O) for 1 hour and washed in ddH_2_O. Then, the samples were dehydrated using a gradient ethanol series, dried and mounted. Finally, the samples were sputter-coated with nanogold. The stereocilia bundles of the cochleae were examined using a Hitachi S-4800 Field-Emission scanning electron microscope.

#### Transmission Electron Microscopy

The basilar membranes were dissected, fixed and dehydrated. To be embedded in resin, ethyl alcohol in tissue was successively replaced with acetone and resin. After this step, tissues were placed into grooves of a mold filled with resin. The mold was transferred into an oven at 50°C overnight. The samples were cut to slices with a thickness of 50nm using a cryo diamond knife and stretched on the water. The collected ultrathin sections were attached to a copper grid and stained with uranyl acetate and lead citrate. Images were acquired using a JEOL-1200 electron microscope. Three KO male mice and two WT male mice were used in this experiment.

#### Noise Exposure

In this study, 1- to 2-month-old male mice were divided into two groups (5 mice per group): a control group (wild-type mice) and an experimental group (*Smpx* knock-out mice). All mice were anesthetized according to the ABR method. Then they were placed in the sound-proof box and exposed to noise limited to the 8-16 kHz octave band at 105 db SPL for 2 h. The auditory brainstem responses of these mice were recorded at four time points: 3 h, 7 days, 14 days, and 30 days after noise exposure. The sound was generated by the noise generator (SF-06, Random Noise Generator, RION, United States), amplified by the power amplifier (CDi 1000 Power Amplifier, Crown, United States), and transmitted to a microphone. The noise files were created and equalized with audio editing software (Audacity Portable). Sound levels were calibrated at multiple locations within the sound chamber to ensure uniformity of the stimulus.

### *In vivo* Transfection by Injectoporation

Plasmid pEGFP-N2 (Clontech) was used as a backbone to express Smpx in living outer hair cells. The whole open reading frame (ORF) was cloned using primers shown in Table 1. pEGFP-N2 and PCR products were digested by *Eco*RI and *Bam*HI (NEB). Mixed products were incubated 10 min at 22°C with T4 DNA ligase (Thermo). Heat inactivation was conducted at 65°C for 10 min. Then, the plasmids were transferred into Trans 5α competent cells. The positive bacteria strains were screened by kanamycin.

Injectoporation was performed as previously described (Xiong et al., 2014). In brief, the otic vesicles from mice between postnatal day 0 (P0) and P8 were dissected from the skull, and the bony capsules were removed and the Corti organ was exposed. The remaining cochlear ducts were transferred to the lid of a 35-mm Petri dish. During the entire procedure, the tissue was covered with a solution to prevent mechanical damage. The tissue was placed on the bottom of the dish attaching to its surface. A microinjection pipette was controlled by a micromanipulator. Smpx-EGFP expression plasmid was injected into the space surrounding the hair cell bodies by a microinjection pipette and the electroporation electrodes were activated. These procedures were controlled by micromanipulators. After overnight culturing, the tissues were examined by an LSM 800 confocal microscope after overnight culture.

## Results

### Clinical Presentation

**FIGURE 1:**
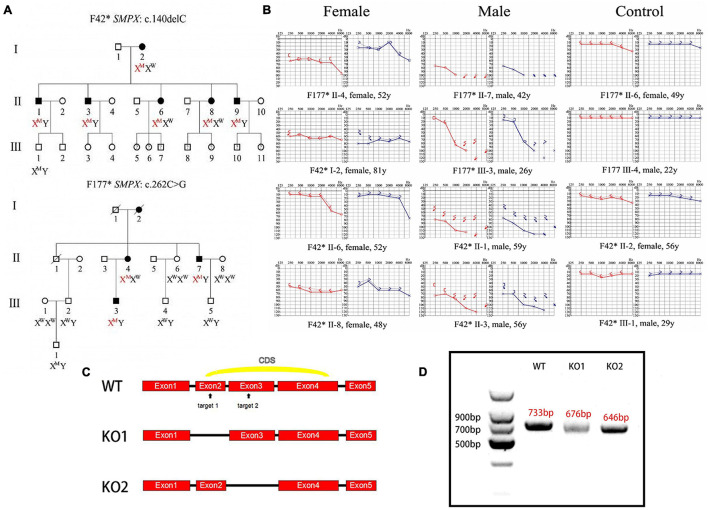
Clinical phenotype and mouse model construction. **(A)** Pedigree of two Chinese families affected by DFNX4. Solid objects mean gene defect; hollow objects mean unaffected members; squares represent males; circles represent females. This pedigree indicated an X-linked dominant inheritance. **(B)** Audiograms of affected family members. Red indicates right ear and blue indicates left ear. We discovered that the hearing threshold of two ears was almost the same except F177* II-4’s; hearing loss was more serious in male than female mice; Hearing loss was significantly worse in high frequency than that in low and medium frequencies and varied among individuals. **(C)** Schematic diagram of Smpx KO mouse construction. Two target sites were chosen in exon 2 and 3 (arrowed); two KO models were obtained. **(D)** Agarose gel electrophoresis of cDNAs acquired from knock-out mice. We cloned the whole Smpx CDS of these two knock-out pattern and discovered 57 and 87 bps deletions, which were exactly the whole length of exons 2 and 3.

In the screened subjects, Two mutations were found to be associated with DFNX4 (SMPX:c.140delC and SMPX:c.262C > G). The patients from family F42* and F177* showed an X-linked non-syndromic sensorineural hearing loss (Figures 1A,B). Due to the limitation of the tested families, only the adult patient information was obtained. A 26-year-old male subject (F177* III-3) suffered a significant high-frequency hearing loss, but had a nearly normal low-frequency hearing. Elderly subjects (F177* II-7, F42* II-1, F42* II-3) exhibited a severer hearing loss at all frequencies. Four female subjects(F42* I-2, F42* II-6, F42* II-8, F177* II-4) exhibited various degrees of hearing loss apparently more mild than males.

### Generation of *Smpx* Knock-Out Mouse

Mouse full length Smpx protein has only 85 aa. The coding sequence (CDS) contains 3 exons (Figure 1C). We chose two target sites in exon 2 and 3, respectively. The mixed mRNA was injected into 100 zygotes of CBA/CaJ background by cytoplasm microinjection. After 2 h of culturing, 50 plump zygotes were transferred into the oviduct ampullary of two pseudopregnancy mothers. Twenty-three fetuses were born (F0) after 19 days of pregnancy, and 11 of them were found with different indels (insertion and deletions) by genotyping. Nine indels were theoretically effective. We then selected 86 base pairs (bps) deletion mutations in the second exon and a 620 bps insertion mutation in the third exon as our experimental subjects (Figure 1C).

To test these knock-out effects, the entire RNA was extracted from the cochlear of knock-out mouse and reversibly transcribed to cDNA (primers were shown in Table 1). cDNAs were cloned into pMD18-T and sequenced. Interestingly, the whole exon 2 in mutation 1 disappeared, and exon 3 vanished in mutation 2. We believed this occurred due to the following reasons. In mutation 1, the deletion led to the loss of the GT-AG splice site, which in turn caused the spicing to skip through the 2nd exon. In mutation 2, the 620bps recombination sequence disrupted the 87bps exon3, which potentially led to the failure to identify the splice site by snRNP. All potential off-target sequences were amplified using primers (Table 2) and sequenced. Fortunately, no mutations on these sites were identified (data not shown).

#### Progressive Hearing Loss in *Smpx* Knock-Out Mice

**FIGURE 2:**
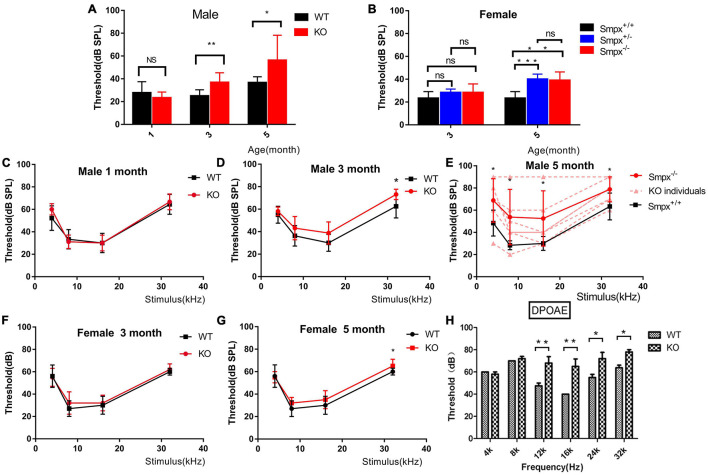
Progressive hearing loss was displayed in ABR and DPOAE. **(A)** Broadband click stimuli induced ABR in male mice. Hearing loss started from the 3rd month and the hearing gradually decreased with aging. **(B)** Broadband click stimuli induced ABR of female mice. Hearing loss started from the fifth month, and homozygous and heterozygous mutation mice displayed similarities with aging. The hearing loss in female mice had a later onset and more mild than that in the males **(C-E)**. Frequency-specific pure tone induced ABR of male mice aged 1, 3, and 5 months. These ABRs indicated that hearing loss started at the 3rd month. The situation deteriorated from high to low frequency. In the 5th month, there was a 20 db threshold shift in 4 kHz pure tone. However, the individual difference was significant shown in panel **(D)**. Two-way ANOVA was used to compare the difference between two groups. *p < 0.05; **p < 0.01; and ***p < 0.001. **(F,G)** Frequency-specific pure tone induced ABR in female mice at the 3rd and 5th month. Compared to male mice, the threshold shift occurred later and was milder. **(H)** DPOAE test on wild type and Smpx KO 3-month-old male mice. Threshold shift appeared from 12 to 32 kHz, which did not appear in low frequency at this time. This result was consistent with that in ABR **(D)**.

Pedigree of X-linked dominant inheritance hearing loss DFNX4 was displayed in Figure 1A. Considering that *Smpx* was an X chromosome-linked gene, *Smpx* KO mice were separated into male and female two groups. We detected auditory brainstem response (ABR) of mice aged 1, 3, and 5 months in both groups (Figure 2). The responses to broadband click stimuli included varying degrees of hearing loss in both male and female mice. The threshold shifts first appeared at a younger age in male mice (3 months) compared to female mice (5 months) (Figures 2A,B). In addition, the male mice showed a greater elevation of mean threshold compared to the mild increase in female mice. These results were consistent with human audiograms which suggested that hearing loss in males was more severe than that in females (Figure 1B). Moreover, threshold shift appeared earlier in high frequency, which was observed from pure tone stimuli ABR (F177* III-3 in Figures 1B, 2C-G). Hearing loss gradually spread from high to low frequency until the whole spectrum was affected by the 5th month. Due to individual difference, all KO curves (dotted line in Figure 2E and the thresholds in detail were shown in Supplementary Table 1A) at 5th month were plotted to show more details of hearing loss in KO mice. We found that both normal hearing and completely deafness accounted for 11% of mice. This conformed to the normal distribution. Mixed effect ANOVAs were performed to evaluate the effect of KO on overall hearing (male click, *p* = 0.0049; female click, *p* = 0.0108) and the difference was significant. We also analyzed the effect sizes of the ABRs with Hedges’ g (Supplementary Table 1B). In brief, the KO caused a medium to large effect on mice hearing from the 3rd month both in male and female mice. In male mice, the KO was effective at all frequencies, whereas it was shown to have a negligible effect at low frequencies in female mice. By comparing effect sizes with audiograms, we found that the KO had already slightly affected the hearing before there was a significant difference (see 3-month female click and 3-month male pure tone).

We also tested DPOAE (Figure 2H) and compared it with ABR. As shown in ABR, hearing loss varied in *Smpx*^–/–^ mice. Some mice normally displayed WT phenotype. However, in DPOAE, the mice concurrently exhibited threshold shifts, which revealed problems in outer hair cells. At the age of 3 months, the tested KO mice showed significant threshold shifts at frequency of 12, 16, 20, and 32 kHz, which represented the middle to high frequency. This result led us to pay more attention to outer hair cells.

### Progressive Degeneration of Stereocilia

**FIGURE 3:**
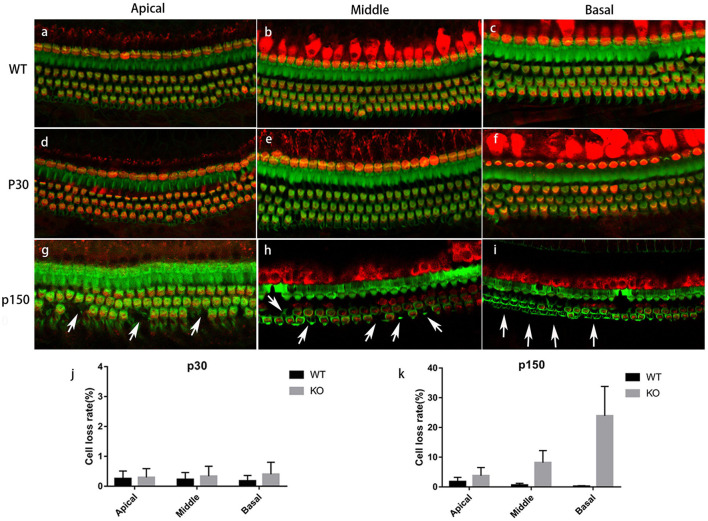
Immunostaining of cochlea basilar membrane. Red: Myo7a, marking the cell bodies; Green: phalloidin, marking the stereocilia. **(a-c)** Basilar membranes from a p150 WT mouse. **(d-f)** Basilar membranes from a P30 Smpx KO mouse. No cell loss was detected, but some OHC bundles were out of shape. **(g-i)** Basilar membrane from p150 Smpx KO mice. Cells loss was sporadically detected at the apical and middle turn, and a large number of cells loss were detected at the basal turn. Scale bar: 20 μm. **(j,k)** OHC loss rate of mice at p30 and p150. This data indicated a progressive OHC loss consisting with the images above.

**FIGURE 4:**
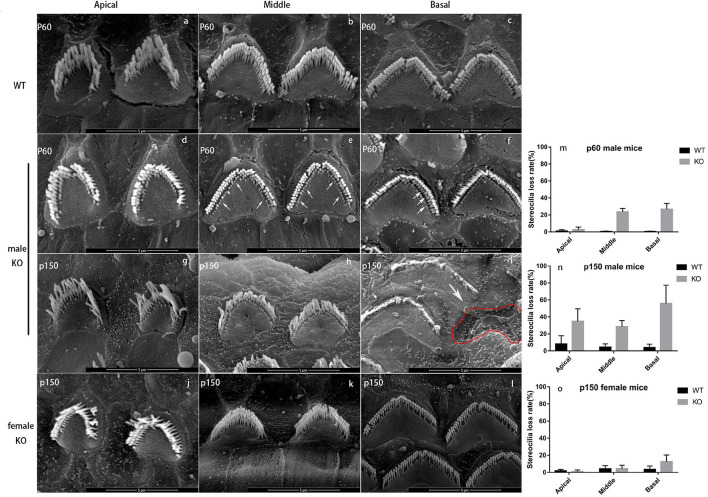
Scanning electron microscope of WT and KO mice. **(a-c)** OHC bundles of wild-type male mouse at p60. There are three rows of stereocilia (“M” shape) on the cuticular plate (heart shape). Scale bar = 5 nm. **(d-f)** OHC bundles of Smpx KO male mouse at p60. The loss of shortest row stereocilia from apex to base showed a trend from less to more (arrowed). However, the middle and longest rows were normal, and the “M” shape was still sharp and clear. **(g-i)** OHC bundles of Smpx KO male mouse at p150. The stereocilia degeneration appeared at the apical turn and the conditions were more severe at the middle and basal turns. The middle and longest rows of OHC stereocilia started to degenerate and the “M” shape was indistinct at the middle turn. The whole bundle degenerated at the basal turn. Some OHCs were dead and left a pit on the basilar membrane (red line). **(j-l)** OHC bundles of Smpx KO female mouse at p150. The bundles were normal in apical and middle turn. A number of the shortest row stereocilia was degenerated at the basal turn. **(m-o)** OHC stereocilia loss rate of p60 male mice, p150 male mice, and p150 female mice. This data indicated a progressive OHC stereocilia degeneration and this process was more mild and had a later onset in female mice.

To determine the changes caused by *Smpx* deficiency, the cochlear hair cell stereocilia bundles were stained with FITC-phalloidin. The cell body was marked by Myo7a (Figure 3). At p30, the OHCs were neatly arranged and no significant cell loss was detected. Nevertheless, abnormal bundles were found on the top of the cuticular plate (Figures 3d-f). At p150, OHC numbers began to gradually decrease (Figures 3g-i). All the above results were confirmed by quantitative analysis (Figures 3j,k). On the basis of this phenotype, a scanning electron microscope was utilized to examine the morphology of the stereocilia in detail (Figure 4).

In male mice, OHC stereocilia degeneration began from p30 and existed only at the end of basal turn near the oval window. The shortest-row of OHC bundles showed a shorter or normal length (data not shown). At p60, most stereocilia in the shortest row of OHC bundles at the basal turn were degenerated. Stereocilia degeneration spread from the base to the apex and some stereocilia in the middle row started to shorten in the basal turn (Figures 4d-f). At p150, OHC stereocilia in the basal turn were usually completely degenerated, or if still present, they were very short and showed signs of fusion (Figure 4i). The shortest row of OHC bundle in the middle and apical turn were mostly degenerated (Figures 4g,h). In female mice, stereocilia degeneration started at p150, and the degeneration process was milder. The shortest row of OHC stereocilia began to degenerate in the basal turn, while those were normal at middle and apical turns (Figures 4j-l). We analyzed this process in a quantitative way by computing the stereocilia loss rate (Figures 4m-o). These results might explain why DFNX4 in females was milder, and the high-frequency hearing deteriorated earlier in both male and female mice.

**FIGURE 5:**
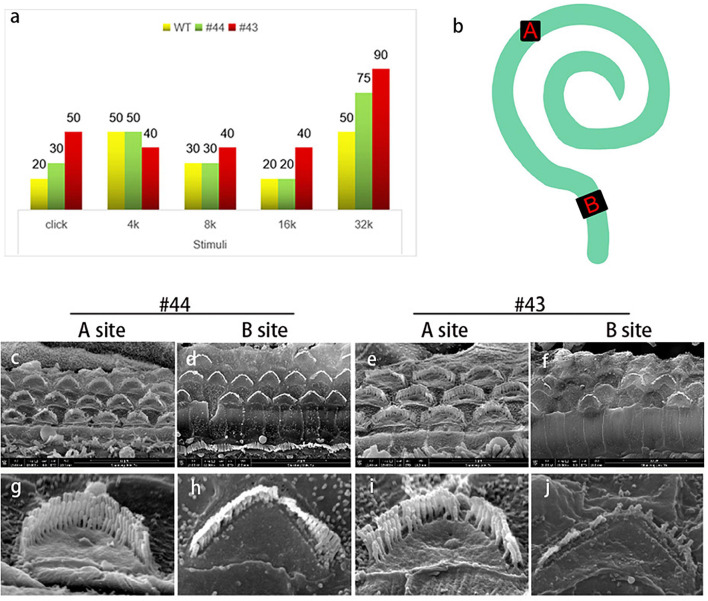
The condition of outer hair cells was closely related to the hearing of the Smpx KO mouse. **(a)** Auditory brainstem response to a pair of sister mice (#43, #44) with different levels of hearing loss. Two mice were both 1-year-old. **(b)** Schematic diagram of the whole basilar membrane. Site A represents the middle turn and responses to medium frequency sound waves; site B represents the basal turn and responses to high-frequency sound waves. **(c-j)** SEM of basilar membranes from the cochlea of #43 and #44 and the amplified image. Comparing **(c,g)** with panels **(e,i)**, middle turn hair bundles in panel **(g)** is normal. The Shortest-row of stereocilia in panel **(i)** starts to degenerate. Reflected in the ABR, there are 10 and 20 dB threshold shifts in #43 at 8 and 16 kHz (medium-frequency), but it is normal in #44. Comparing **(d,h)** with panels **(f,j)**, stereocilia degeneration in #43 is badly worse than that in #44. Large numbers of hair cells’ death in panel **(f)** resulted in the loss of hearing in a high frequency of #43. The remaining hair cells and stereocilia ensured that #43 maintained a low-level hearing in high frequency.

Although OHC stereocilia showed progressive degeneration, no changes were observed in IHC stereocilia compared to WT mice. To confirm whether OHC stereocilia degeneration was consistent with hearing loss and whether the remaining stereocilia were still sensitive to the vibration, a pair of sister mice’s data (#43,#44) were individually shown (Figure 5). ABR was tested 1 day before the basilar membrane was dissected and observed under SEM. We chose two locations as the targets (Figure 5b). Site A was on the middle turn of the basilar membrane, responding to the medium frequency hearing. Site B was on the basal turn, responding to the high-frequency hearing. Broadband click stimuli ABR suggested that these subjects had a varing degree of hearing loss (Figure 5a). For #44 (Figures 5c,d,g,h), stereocilia in site A were intact and straight. In site B, some OHCs were lost, and a whole row of stereocilia disappeared in the remaining cells. These changes were reflected in the ABR thresholds. Low and medium frequencies hearing were normal, while there was a 25 dB threshold shifted in 32 kHz. For #43 (Figures 5e,f,i,j), some stereocilia degenerated in site A. In site B, large numbers OHCs were lost, and only some longest-row stereocilia remained. These changes were reflected in the ABR thresholds. 8-16 kHz hearing had a 10-20 dB threshold shift and hearing in 32 kHz was nearly lost. These evidence supported the observations from DPOAEs and the affected OHCs were likely to be largely responsible for the hearing impairment.

### FM1-43 Uptake

**FIGURE 6:**
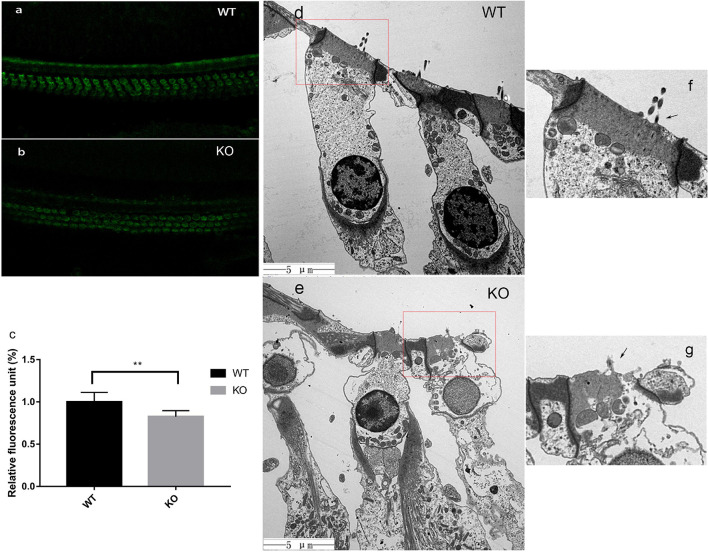
In KO mice, the FM1-43 uptake decreased and cell edema began to appear at the end phase of stereocilia degeneration. **(a,b)** FM1-43 uptake in hair cells of WT and Smpx KO mouse at p60. This image indicated that the shortest row stereocilia degeneration did not greatly affect the uptake of FM1-43. **(c)** relative fluorescence unit of FM1-43 uptake. The uptake in KO mice at p60 was reduced by 17%. **(d,e)** Transmission electron microscope of hair cells in WT and Smpx KO mouse at p150. In panel **(e)**, the cellular contents were disturbed by water, and swelling appeared on the surface of cell membrane. Undoubtedly, these were characteristics of cell edema. This state of hair cells was more sensitive. **(f,g)** Magnified image of panels **(d,e)**. In panel **(g)**, the longest row was still remaining (arrowed), which indicated that cellular edema occurred during the stereociliary degenerating process but not after the process. ** means 0.01 > p value > 0.001.

Previous studies have reported that the mechanically gated ion channel is on the top of stereocilia and located at the lower tip-link end (Beurg et al., 2009). To explore whether the MET channel was still present, an simple assay for FM1-43 uptake was performed in the hair cells of 2-month-old mice (Figures 6a,b). A clear fluorescence dye uptake was observed in WT and KO living hair cells. The relative fluorescence unit was computed by the ImageJ software. The results indicated that ion channel decreased to some extent with statistical difference (Figure 6c). We describe earlier that middle turn OHCs of 2-month-old mice were still present, although their shortest row of stereocilia had degenerated (Figures 4d-f). Combined with the results of the FM1-43 assay, it was likely that the remaining stereocilia still maintained a mechanical-electrical transduction function.

### Cell Death in *Smpx*-Null Mice

At the 5th month, a large number of OHC deaths, especially at the basal turn of the basilar membrane, were observed in *Smpx*-null mice. With aging, cell death spread from the base to the apex, and outer hair cell survival at the basal turn was decreased. Based on the fact that OHC death came later than stereocilia degeneration, we assumed that OHC death might be secondary to stereocilia degeneration.

We used TEM to examine the condition of the outer hair cell body after stereocilia degeneration (Figures 6d,e). At 5000X, we discovered an obvious transformation corresponding to the characteristics of cellular edema (Xu et al., 2017). There were some substantial edematous changes in the organelles and vacuolation of the mitochondria. The cellular contents were disturbed by water, and swelling appeared on the surface of the membrane. Remarkably, this phenomenon in the cell body had already taken place before stereocilia completely degenerated (Figures 6f,g). The cell body was filled with water, while the stereocilia were still present (Figure 6g). These data suggested that stereocilia degeneration might be linked to cellular swelling and final cell death.

#### Localization of *Smpx* in Hair Cells

**FIGURE 7:**
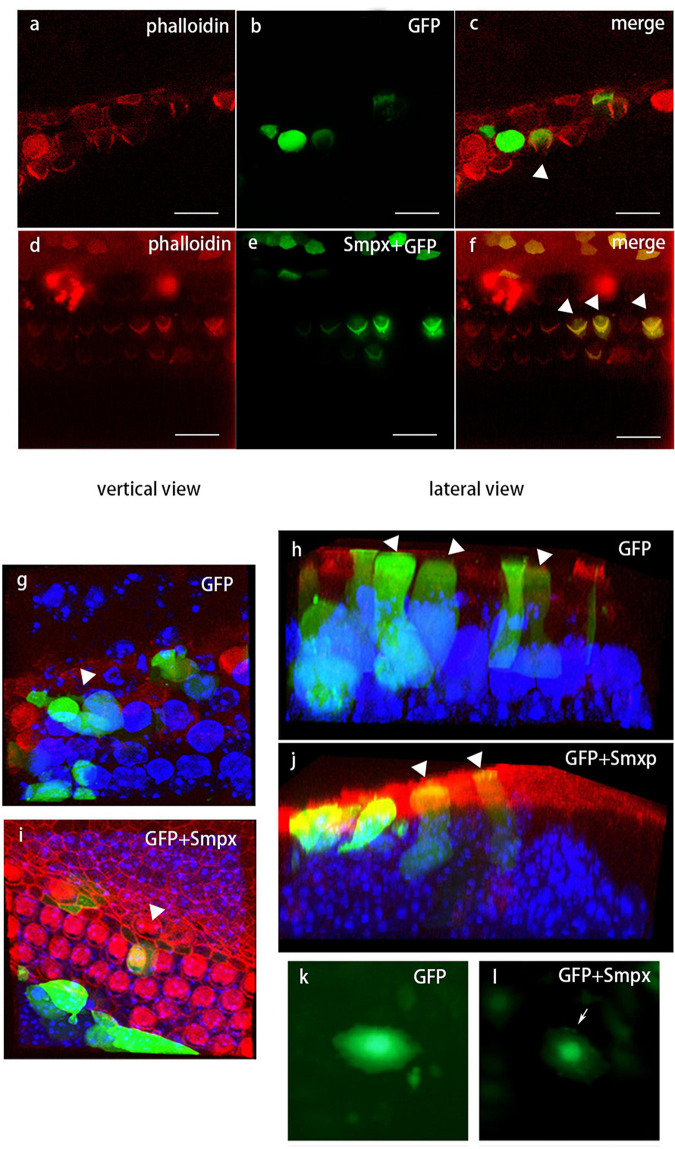
Localization of *Smpx* in hair cells *via in vivo* electrotransfection (injectoporation). Red, phalloidin; Green, GFP. **(a-c)** Hair cells transfected with pEGFP. These images showed GFP was expressed in the cell body but not in the stereocilia. **(d-f)** Hair cells transfected with pEGFP-Smpx. The obvious “V” shape indicated that GFP-Smpx was located in stereocilia. **(g-j)** Three-dimensional image of transfected hair cells. Red, phalloidin; Green, GFP; blue, DAPI. 3D images displayed a clearer expression of GFP and GFP-Smpx. **(k,l)** Hela cells transfected with pEGFP and pEGFP-Smpx. The fusion protein GFP-Smpx should be tested before injectoporation to confirm if GFP would affect the localization of Smpx.

To verify the localization of Smpx in outer hair cells, we tried nearly all the commercial antibodies against Smpx, but none of them worked well. Instead, we used injectoporation to deliver pEGFP and pEGFP-Smpx plasmids to mechanosensory hair cells (Figure 7). Smpx was made of 85 amino acids. In order to test if the EGFP would interrupt the localization of Smpx, the plasmids were first transfected into Hela cells (Figures 7k,l). GFP-Smpx were concentrated in the nuclear skeleton and lamellipodia (arrowed) where actin was found, while GFP was diffusely located throughout the cytoplasm. After being delivered into outer hair cells by injectoporation, an obvious expression along the whole cell body was detected in the GFP and Smpx + GFP groups. Although GFP was abundantly expressed in the cell body, no signal was detected on bundles (Figure 7b). On the contrary, an obvious “V” shape on the cuticular plate was observed, indicating that Smpx-GFP was located in stereocilia (Figure 7e).Three-dimensional images displayed a clearer expression of GFP and Smpx + GFP throughout the cell (Figures 7g-j). Localization of Smpx to the OHC stereocilia provided good evidence that Smpx-deficiency was closely linked with stereocilia degeneration. In addition, this experiment confirmed the expression of Smpx in stereocilia, but could not exclude its existence in the cytoplasm.

### Noise Exposure

We found that mice within 3 months of age had a normal hearing, but the stereocilia had already degenerated at p60. To examine whether hearing of KO mice was more vulnerable to loud sound under 3 months. we exposed two groups of 1- to 2-month-old mice (WT and KO mice) to loud noise (Supplementary Figure 1). Both groups showed 20-30 dB threshold shift in ABR after 2 h of noise exposure. Thresholds of WT mice recovered after one week and thresholds of KO mice did not recover, which suggested that noise-exposure had induced a permanent injury to the outer hair cells of the KO mice. The unrecoverable hearing loss in KO mice indicated that the absence of Smpx had already affected the mice hearing within 3 months of age. It took some intense stimulation for the influence to show up. These results suggested that the DFNX4 patients should avoid excessively loud sound exposure as much as possible.

## Discussion

Sensorineural hearing loss affects nearly 300 million individuals. It mainly arises from damage or death of auditory hair cells (Liu et al., 2016; Liu W. et al., 2019; Gao et al., 2020; Chen et al., 2021; He et al., 2021) and spiral ganglion neurons (SGN) (Liu Y. et al., 2019; Ding et al., 2020; Guo et al., 2020; Liu et al., 2021; Yuan et al., 2021), which can be caused by environmental insults (such as overexposure to loud sounds or exposure to aminoglycoside antibiotics or chemotherapeutics) (He Z. et al., 2017; He et al., 2020; Gao et al., 2020; Jiang et al., 2020; Zhang et al., 2020; Zhong et al., 2020; Zhou et al., 2020), or genetic alteration (He et al., 2017; Fang et al., 2019; Qian et al., 2020; Fu et al., 2021; Lv et al., 2021; Zhang et al., 2021). Sound is collected and conducted by the external and middle ear, and transformed into electrical signals by cochlear inner and outer hair cells, Then the electrical signals are transduced to the auditory cortex through spiral ganglion neurons (Martin et al., 2003; Sun et al., 2016; Guo et al., 2019, 2021; Zhao et al., 2019; Hu et al., 2021). *SMPX* is closely linked to sex-linked non-syndromic hearing loss DFNX4. It was first identified and mapped in 1999. Within the 88 amino acids of SMPX protein, the predicted peptide showed no significant homologies to known structural elements (Patzak et al., 1999). Previous studies have reported that mutations in *Smpx* cause progressive autosomal recessive non-syndromic deafness DFNX4. The *Smpx* deficient mice made on C57BL/6 background showed no obvious phenotype (Huebner et al., 2011; Schraders et al., 2011; Abdelfatah et al., 2013). Therefore, this mice strain was not further preserved.

In this study, we established and validated Smpx KO mice using CRISPR/Cas9-mediated genomic editing technology in CBA/CaJ mice to explore the function of Smpx and the underlying mechanism its action in the hearing system. With the cooperation of the Institute of Otolaryngology, West Hospital of Shandong Provincial Hospital, we also obtained some patients data and new mutation sites of DFNX4, which had never been reported. The male subjects displayed a more severe hearing loss than the female subjects and high frequency hearing seemed worse in both males and females. To confirm whether our mouse model was suitable, we tested the ABR of *Smpx* KO mice. In male mice, definite hearing loss started from the 3rd month for high frequencies and gradually increased with time. Female mice had a milder hearing impairment and developed hearing loss later than male mice. This progressive hearing loss process was highly consistent with that in DFNX4 patients, which suggested that Smpx KO mouse could be used as an mouse model for DFNX4 research.

The deficiency of some actin binding proteins expressed in stereocilia can lead to the shortening or even degeneration of stereocilia. As an actin binding protein, Smpx expression site is essential in determining whether Smpx is functional in stereocilia. The most common way to prove the localization of Smpx in hair cells is the positive staining using a corresponding antibody. Unfortunately, we didn’t find a suitable commercial antibody that worked. Instead, we used injectoporation to deliver a plasmid containing an ORF of Smpx binding with EGFP into living hair cells. After 24 hours of culture and expression, we detected the fluorescence signal of Smpx-EGFP in stereocilia and cell body, while EGFP was only expressed in the cell body. The null mutation of Smpx led to stereocilia degeneration after p60. Since Smpx was localized on stereocilia, we speculated that Smpx might play a role in maintaining OHC bundles.

At the age of 2 months, stereocilia of KO male mice started to degenerate from the shortest row of OHC bundles. Also at the age of 2 months, FM1-43 uptake in OHCs of KO mice was a little lower than that of WT mice, but the hair cells were still positively stained. This result implied that degeneration of the shortest row stereocilia in OHC bundle didn’t greatly affect ion channel. As a simple way, FM1-43 uptake was an imperfect method to test the MET channel. Yet, these findings still gave us a preliminary indication whether MET channel function was affected.

### *Smpx* Knock-Out Mouse Is an Ideal Model for Examining DFNX4 and Stereocilia Degeneration

Animal models are extremely important for investigation of the roles played by genes in the hearing process. The genetic background of mice sometimes affects the phenotype of mutant mice. Thus, it is crucial to use an appropriate animal strain with regard to a specific research field such as hearing. For example, C57BL/6 mice carry the Ahl allele of Cdh23 which predisposes them to early onset presbycusis and is therefore not always an ideal choice for auditory research. In a previous study (Palmer et al., 2001), *Smpx* KO mouse models with age-related hearing loss (ARHL) were generated by gene-targeting based on C57/BL6 background. We could not tell if the ARHL was caused by genetic mutation or mouse background. In this study, no obvious abnormities were found in mice and the inbreeding of mice strain was discontinued (confirmed by e-mail). CBA/CaJ mouse without ARHL is a better choice in hearing research especially when studying progressive hearing loss.

Comparing the audiograms, we showed that our mouse model could mimic the progressive hearing loss observed in humans. To date, no mice cochleae with *Smpx* mutation have been histologically studied. Using the *Smpx*-null mouse model, we investigated the degeneration of OHC stereocilia bundles and the resulting effect on Smpx function in the outer hair cells. With the OHC stereocilia degeneration mouse model, we can further study OHC bundle structures, such as MET channel and tip-link. Moreover, using SEM, we found the degeneration of OHC stereocilia at the shortest row in the 2nd month. In the test of FM1-43 uptake, there was a 17% signal reduction in KO OHCs which indicated the weakened function of MET channel. Interestingly, the hearing loss started from the 3rd month. For a further research, we put the WT and KO mice (under 2 months of age) exposed to a certain intensity noise. The WT mice recovered after a week as expected while the KO mice suffered a permanent injury to their hearing. In a conclusion, though young mice under 2 months of age had a normal hearing, they were more sensitive to loud noise. This gave us a hint that, DFNX4 patients were more susceptible to noise and they should stay away from excessively loud noise to avoid serious hearing loss.

### *Smpx* May Play a Role in Maintaining Hair Cell Bundles

In OHCs, the stereocilia contain a core of actin filaments associated with actin binding proteins. As an ABP, Smpx was also expressed in OHC stereocilium. Degeneration of the OHC stereocilia and the unrecovered hearing after noise exposure gave us a hint that Smpx might play a role in maintaining the hair cell bundles. In the previous study (Palmer et al., 2001; Eftestøl et al., 2014), Smpx was shown to be expressed in pleural segment muscle where its functions included binding the sarcomere to cytomembrane. The localization where Smpx expressed in muscle cells could be described as the bridge rib, which not only connected but also supported the sarcomere (actin filament) to the cytomembrane. We will make a assumption that Smpx may have a similar role in hair bundles, i.e., it may provide a supporting link between the actin core and surrounding structures.

## Data Availability Statement

The original contributions presented in the study are included in the article/Supplementary Material, further inquiries can be directed to the Corresponding Author/s.

## Ethics Statement

The studies involving human participants were reviewed and approved by Ethics Committee of the Shandong University. The patients/participants provided their written informed consent to participate in this study. The animal study was reviewed and approved by Ethics Committee of the Shandong University.

## Author Contributions

JG and HW supervised the project. HW and XB provided the information of human DFNX4 patients. AZ, HT, and XF generated the *Smpx* KO mouse model. HT and SX performed the statistical analysis and acquired the data. HT performed the experiment and analyzed and interpreted the results. HT and JG wrote the manuscript. All authors approved the submitted version.

## Conflict of Interest

The authors declare that the research was conducted in the absence of any commercial or financial relationships that could be construed as a potential conflict of interest.

## Publisher’s Note

All claims expressed in this article are solely those of the authors and do not necessarily represent those of their affiliated organizations, or those of the publisher, the editors and the reviewers. Any product that may be evaluated in this article, or claim that may be made by its manufacturer, is not guaranteed or endorsed by the publisher.

## Footnotes

1
https://hereditaryhearingloss.org/xlinked-genes

2
https://cm.jefferson.edu/Off-Spotter/

## Supplementary Material

Supplementary Figure 1 Noise exposure of mice less than 2 months old. The KO mice suffered permanent hearing impairment, while the WT mice recovered in a week.

Supplementary Table 1 Thresholds of KO male mice at 5 months of age and the effect size of ABRs.
