# Supplementary figures and images for: SMPX Deficiency Causes Stereocilia Degeneration and Progressive Hearing Loss in CBA/CaJ Mice
Source: Front Cell Dev Biol. 2021 Oct 14;9:750023. doi: 10.3389/fcell.2021.750023 (PMC8551870; doi:10.3389/fcell.2021.750023)

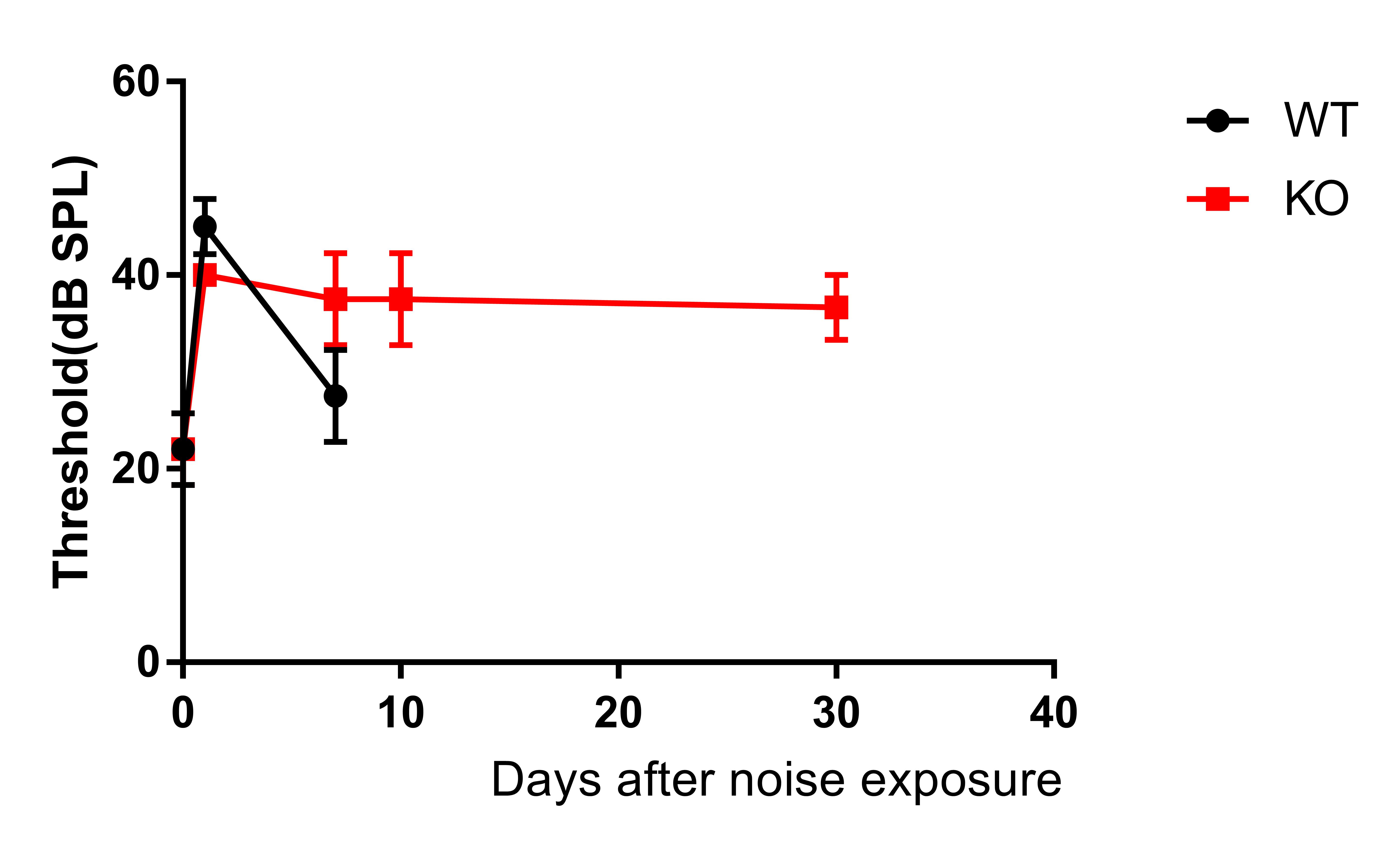

Supplement: Supplementary Figure 1 — Noise exposure of mice less than 2 months old. The KO mice suffered permanent hearing impairment, while the WT mice recovered in a week. [file Image_1.JPEG]
