# Supplementary material for: SMPX Deficiency Causes Stereocilia Degeneration and Progressive Hearing Loss in CBA/CaJ Mice
Source: Front Cell Dev Biol. 2021 Oct 14;9:750023. doi: 10.3389/fcell.2021.750023 (PMC8551870; doi:10.3389/fcell.2021.750023)
Supplement: Supplementary Table 1 — Thresholds of KO male mice at 5 months of age and the effect size of ABRs. [file Table_1.DOC]

| Tone burst frequency(kHz) | Thresholds of KO male mice at 5 months age | | | | | | | |
| --- | --- | --- | --- | --- | --- | --- | --- | --- |
| 4 | 70 | 60 | 70 | 60 | 90 | 90 | 80 | 30 |
| 8 | 60 | 40 | 50 | 40 | 90 | 90 | 40 | 20 |
| 16 | 60 | 30 | 40 | 40 | 90 | 90 | 40 | 30 |
| 32 | 90 | 70 | 80 | 80 | 90 | 90 | 70 | 60 |

a

b

|  |  |  |  | **g** | **95% CI** |  |
| --- | --- | --- | --- | --- | --- | --- |
| Male mice click | | WT vs KO | 1 month | -0.408 | -1.217 - 0.4 | Small effect |
|  |  |  | 3 months | 1.683 | 0.543 - 2.824 | Large effect |
|  |  |  | 5 months | 1.143 | 0.003 - 2.283 | Large effect |
| Female mice click | | WT vs Smpx ^+/-^ | 3 months | 0.775 | -0.399 - 1.948 | Medium effect |
|  |  |  | 5 months | 2.165 | 0.673 - 3.657 | Large effect |
|  |  | WT vs Smpx ^-/-^ | 3 months | 0.775 | -0.399 - 1.948 | Medium effect |
|  |  |  | 5 months | 2.5 | 0.919 - 4.082 | Large effect |
|  |  | Smpx ^+/-^ vs Smpx ^-/-^ | 3 months | 0 | -1.132 - 1.132 | trivial |
|  |  |  | 5 months | -0.114 | -1.354 - 1.127 | trivial |
| Male mice  tone  burst | 1 month | WT  vs  KO | 4k | 0.915 | -0.056 - 1.886 | Large effect |
|  |  |  | 8k | -0.298 | -1.227 - 0.631 | Small effect |
|  |  |  | 16k | 0 | -0.924 - 0.924 | trivial |
|  |  |  | 32k | 0.278 | -0.65 - 1.206 | Small effect |
|  | 3 months |  | 4k | 0.525 | -0.472 - 1.521 | Medium effect |
|  |  |  | 8k | 0.704 | -0.306 - 1.714 | Medium effect |
|  |  |  | 16k | 0.993 | -0.046 - 2.031 | Large effect |
|  |  |  | 32k | 1.327 | 0.245 - 2.41 | Large effect |
|  | 5 months |  | 4k | 1.218 | 0.067 - 2.369 | Large effect |
|  |  |  | 8k | 1.317 | 0.151 - 2.482 | Large effect |
|  |  |  | 16k | 1.155 | 0.014 - 2.297 | Large effect |
|  |  |  | 32k | 1.327 | 0.16 - 2.494 | Large effect |
| Female mice  tone burst | 3 months |  | 4k | -0.11 | -1.159 - 0.938 | trivial |
|  |  |  | 8k | 0.579 | -0.49 - 1.649 | Medium effect |
|  |  |  | 16k | 0 | -1.048 - 1.048 | trivial |
|  |  |  | 32k | 0 | -1.048 - 1.048 | trivial |
|  | 5 months |  | 4k | -0.126 | -1.175 - 0.922 | trivial |
|  |  |  | 8k | 0.822 | -0.269 - 1.913 | Large effect |
|  |  |  | 16k | 0.625 | -0.448 - 1.698 | Medium effect |
|  |  |  | 32k | 1.054 | -0.064 - 2.172 | Large effect |

g: Hodges’ g 95% CI：95% Confidence interval

g<0.2, trivial; 0.2<g<0.5, small effect; 0.5<g<0.8, medium effect; g>0.8,large effect. The negative signs refer to that the mean of KO group is less than the mean of WT group.
